# Avoid Falsely Accusing Female Athletes Who Use Levonorgestrel of Doping

**DOI:** 10.1002/dta.3925

**Published:** 2025-07-10

**Authors:** Alexander Andersson, Anton Pohanka, Mikael Lehtihet, Lena Ekström

**Affiliations:** ^1^ Department of Laboratory Medicine Karolinska Institutet Stockholm Sweden; ^2^ Department of Clinical Pharmacology Karolinska University Hospital Stockholm Sweden; ^3^ Department of Medicine Karolinska Institutet Stockholm Sweden

## Abstract

Athletes are explicitly responsible for everything they consume, which may be an issue when the metabolic pathways of prohibited and non‐prohibited compounds intersect. This was the case when 18‐methyl‐19‐noretiocholanolone, an 18‐methyl‐19‐nortestosterone metabolite, was detected in a sample of an athlete that had used an emergency contraceptive pill containing levonorgestrel.

Six women were recruited to this study to elucidate the link between 18‐methyl‐19‐noretiocholanolone and levonorgestrel. After providing a pre‐treatment urine sample, one tablet of NorLevo, 1.5 mg, was ingested and six additional urine samples were collected. The samples were analysed with GC–MS/MS after extraction and derivatisation.

In all six participants, 18‐methyl‐19‐noretiocholanolone could be detected at 1.5–2.5 ng/mL with a t_max_ of 2 h. The presence of 18‐methyl‐19‐noretiocholanolone was in all samples accompanied by levonorgestrel and its metabolite tetrahydronorgestrel, the latter being present at highest concentrations (60–300 ng/mL) up to 48 h post intake.

Conclusively, this study demonstrates a metabolic link between 18‐methyl‐19‐noretiocholanolone and levonorgestrel, confirming the need to verify the absence of levonorgestrel or its markers before reporting an adverse analytical finding.

## Graphical abstract

Levonorgestrel, a hormonal contraceptive, metabolises to 18‐methyl‐19‐noretiocholanolone which is also the main metabolite of the prohibited substance 18‐methyl‐19‐nortestosterone. Analysing for levonorgestrel and/or tetrahydronorgestrel is essential to avoid misinterpreting 18‐methyl‐19‐noretiocholanolone findings.

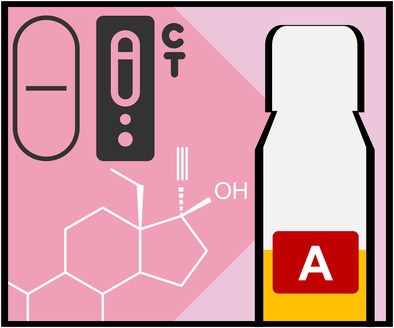

## 1 Introduction

Today's athletes require intimate knowledge of the World Anti‐Doping Code, which is regularly updated by the World Anti‐Doping Agency (WADA). The code outlines the rules as they pertain to doping control and includes, for example, whereabouts tracking and prohibited compounds [1]. The code explicitly expresses that athletes are strictly liable for any findings in the collected samples [2].

**FIGURE 1:**
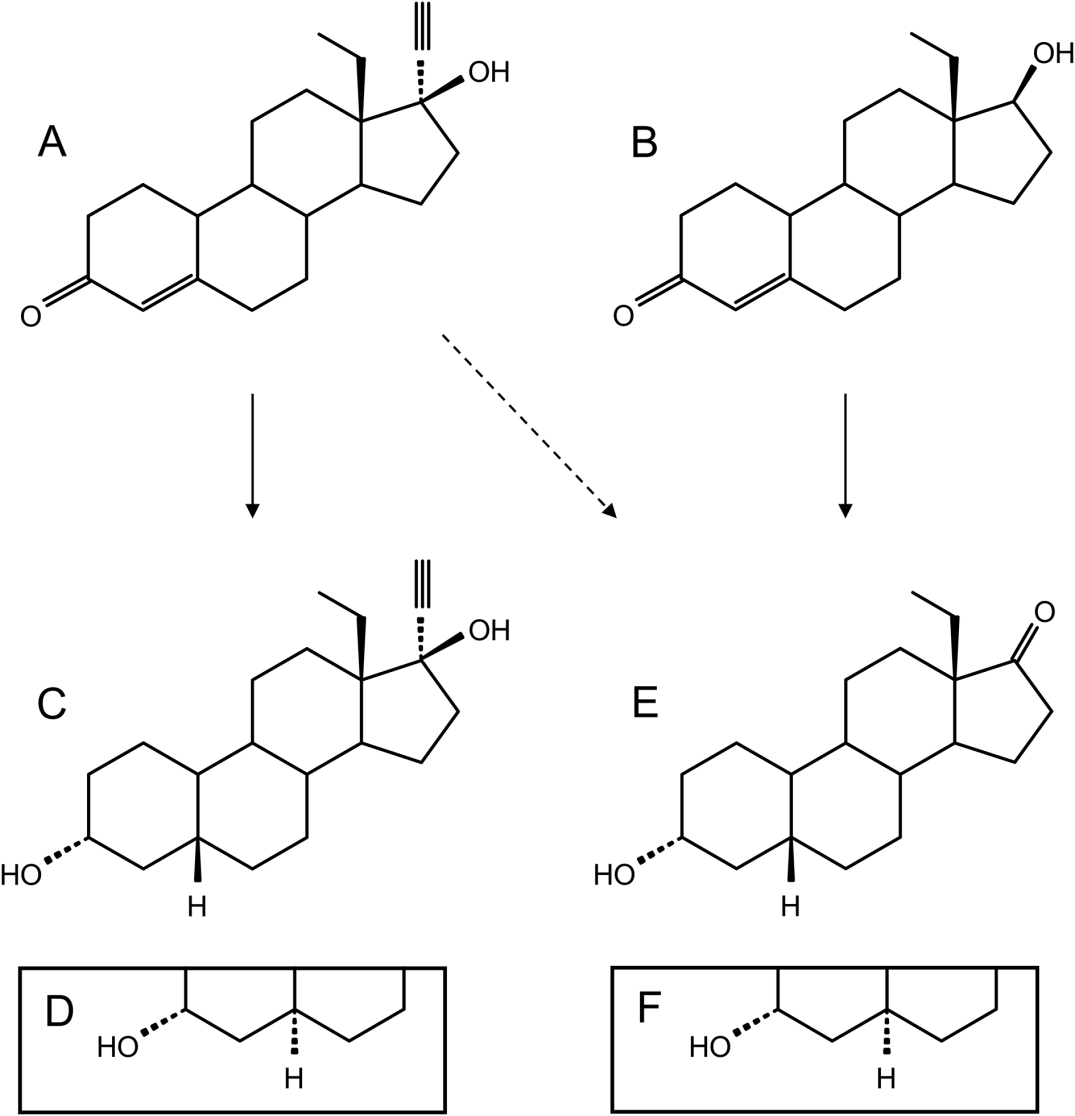
*A schematic drawing of the compounds of interest. The dashed line represents the potential adverse transformation. **A**: Levonorgestrel (LNG), **B**: 18‐methyl‐19‐nortestosterone, **C**: 5*β*‐tetrahydronorgestrel, **D**: 5*α*‐tetrahydronorgestrel (THNG), **E**: 18‐Me‐19‐noretiocholanolone (MNE), **F**: 18‐Me‐19‐norandrosterone. 18‐Me‐19‐norepiandrosterone (MNEA), used as a surrogate for calibrating, is the 3*β*‐hydroxy configuration of MNE (E)*.

Hormonal contraceptives (HC) are widely used by women, including athletes [3, 4] and are available in various formulations. They have been shown to interfere with doping control analysis in some cases [5]. Norethisterone is an active pharmaceutical ingredient in some HC and is not recommended for athletes because of its biotransformation into the prohibited compound 19‐norandrosterone [6, 7]. A related compound, levonorgestrel (LNG), has a homologous structure to norethisterone with an additional methylation of the 18‐carbon. LNG was first studied in the 1960's and was commercially available shortly thereafter [8, 9]. It is used at low doses as prophylactic birth control and at higher doses (× 10) as an emergency HC [10]. Early in vivo studies identified the main metabolite to be tetrahydronorgestrel (THNG) [11, 12]. See Figure 1 for structural detail.

During routine analysis at the Karolinska doping control laboratory, a presumptive adverse analytical finding for the metabolites of the prohibited anabolic steroid 18‐methyl‐19‐nortestosterone (18MENT) was determined, Figure 1 [13]. The athlete had declared the use of an emergency HC on the doping control form. This led to an investigation that eventually resulted in dismissing the case. Related studies have recently been published by the Institute of Doping Analysis and Sports Biochemistry in Dresden, where the routine analysis of an athlete sample detected the same prohibited metabolites [14]. In their investigation, a dietary supplement was found to contain the designer steroid methoxydienone. This shows the significance of still targeting this group of steroids in doping control analysis today.

This study is aimed to solidify the relationship between the LNG and 18MENT metabolites, Figure 1. Our hypothesis is that the excretion of LNG itself and its presumptive metabolite THNG can be used to confirm a legitimate source of the prohibited metabolite 18‐methyl‐19‐noretiocholanolone (MNE).

## 2 Materials and Methods

### 2.1 Study Design

This study was approved by the local ethics committee in Stockholm (2013/720–31/4) with an amendment approval from the Swedish ethics review authority (2022‐04589‐02‐274831).

**FIGURE 2:**
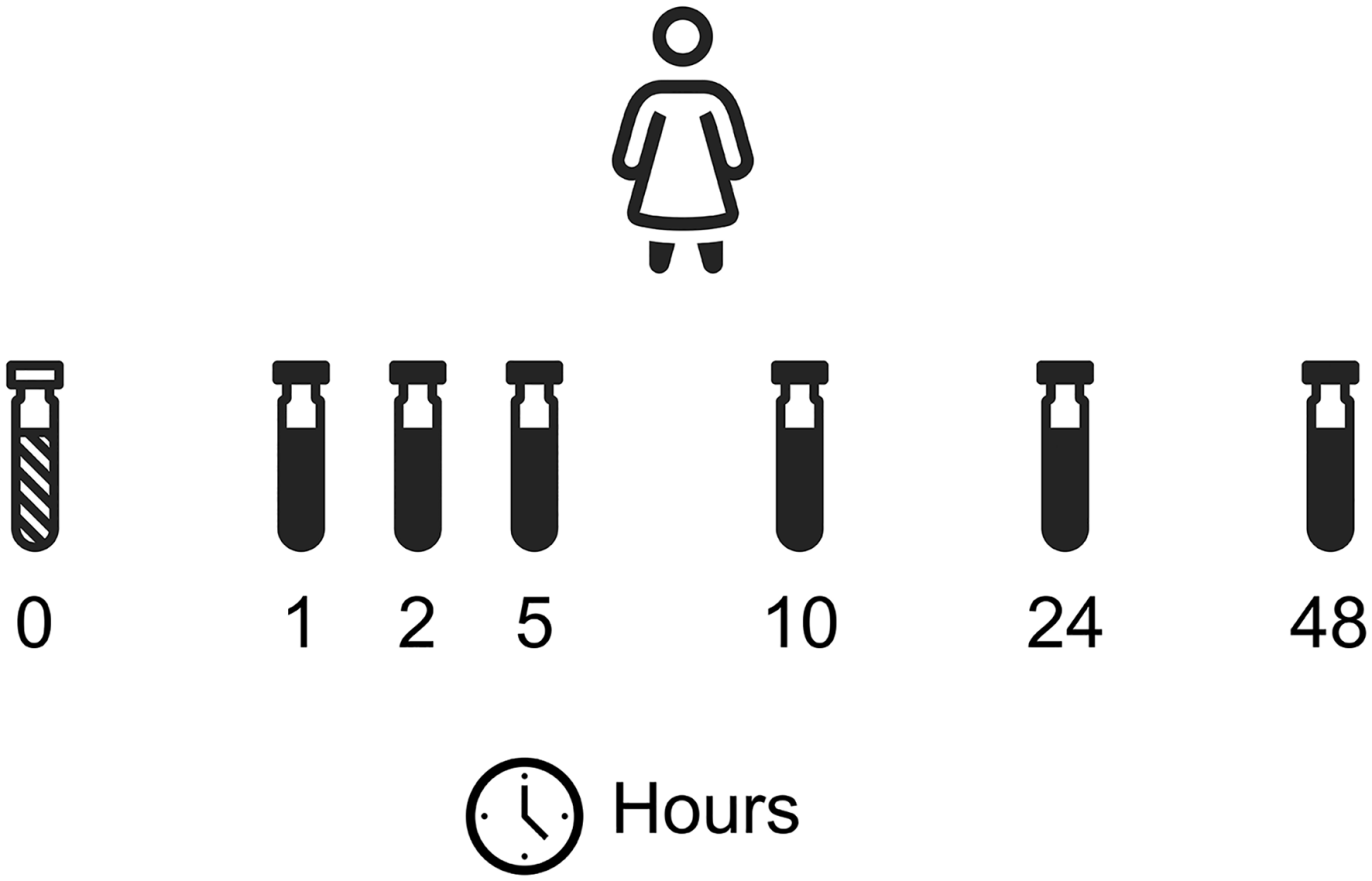
Urine sample collection interval for all participants. NorLevo was administered immediately after t_0_.

Informed written and verbal consent was collected from all participants. Subjects were asked to complete a health survey to ensure that no underlying risk factors were present. Inclusion criteria were as follows: females of age 18–50 with a regular menses, blood pressure below 140/90, not currently using any HC, no known hereditary thrombogenicity and a negative pregnancy test. At inclusion, the subjects were handed seven pre‐labelled urine collection vessels, a sample collection protocol, and a single dose of NorLevo, 1.5 mg, (Laboratorie HRA Pharma, Chatillon, France). Urine samples from five subjects were collected. A baseline sample was collected (t_0_) immediately prior to administration, followed by six urine samples collected as outlined in Figure 2.

### 2.2 Material

18‐methy‐19‐norepiandrosterone (MNEA) was purchased from Atlanchim Pharma (Saint‐Herblain, France). Levonorgestrel, 18MENT and THNG were purchased from Toronto Research Chemicals (Toronto, Canada). d3 epitestosterone was purchased from LGC standards (Teddington, England).

Potassium phosphate buffer, 1.1 mol/L, pH 6.7 was ordered from internal hospital services. Sodium carbonate, sodium sulphate, ethanethiol, ammonium iodine and acetone were purchased from VWR (Radnor, USA). β‐glucuronidase was purchased from Merck (Darmstadt, Germany). Tert‐butyl methyl ether was purchased from Fisher Chemical (Hampton, USA). N‐methyl‐N‐(trimethylsilyl)trifluoroacetamide (MSTFA) was purchased from Larodan (Solna, Sweden).

Derivatisation reagent mixture was mixed by adding ammonium iodine and ethanethiol to MSTFA in the proportions 2:3:1000 (W/V/V).

### 2.3 Sample Preparation

18‐methyl‐19‐norepiandrosterone was used as a surrogate compound for MNE throughout the method since MNE was not commercially available.

The preparation method used was an in‐house WADA accredited initial testing procedure using gas‐chromatography coupled with tandem mass spectrometric detection. It has previously been described by Mullen et al. with a modification to the derivatisation procedure [15, 16]. In short, 50 μL β‐glucuronidase from *E. coli* , 500 μL 0.1 M potassium phosphate (pH 6.7) and internal standard were added to 2 mL urine and incubated for 60 min at 50°C. After cooling to room temperature, 250 μL aqueous potassium carbonate (20% W/V) was added, followed by 5 mL methyl *tert‐*butyl ether and 0,25 g sodium sulphate. The samples were then shaken for 10 min, followed by 5 min centrifugation at 800 g. The organic solvent was transferred to fresh tubes and dried. The sample was transferred to injection vials using 250 μL acetone and evaporated to dryness. 40 μL derivatisation reagent was added and the samples were capped. After incubation for 30 min at 80°C, the vials were ready for analysis.

### 2.4 Instrumentation

Three microliter of sample was injected on an Agilent 7000C GC–MS/MS instrument equipped with a HP‐Ultra 1; 17 m × 0.2 mm with a 0.11 μm film. The inlet and transfer liner were set to 280°C. The temperature program applied was as follows: 180°C, 231°C at 3.3°C/s, 310°C at 30°C/s and held for 2 min. The mass spectrometric conditions and targets can be seen in Table 1.

**TABLE 1:** Mass spectrometric parameters and calibration ranges used. The qualifying transition was used once per participant to verify peak identity, after which retention time was used.

Target	Transition	ce	RT	Range
	SRM	eV	min	ng/mL
Levonorgestrel	316.0 > 194.0 456.0 > 301.2	10 23	14.5	0.5–6.0
18‐methy‐19‐norepiandrosterone/18‐methy‐19‐noretiocholanolone	405.0 > 225.0 405.0 > 315.0	15 10	10.2 (9.8)	0.5–6.0
Tetrahydronorgestrel	459.8 > 417.5 459.8 > 246.7	5 15	12	11–130
18‐methyl‐19‐nortestosterone	431.9 > 301.3 431.9 > 193.9	5 20	12.4	0.5–6.0

Specific gravity (SG) was measured using an ATAGO UG‐1 digital refractometer.

### 2.5 Assay Performance

The following parameters were assessed for LNG, MNEA, THNG and 18MENT: linearity, precision and accuracy at five levels using single replicates over 5 days. Selectivity was assessed by analysing 10 representative urine samples. Matrix effect was tested by analysing accuracy across six representative urine samples at the lower limit of quantification and upper limit of quantification (ULOQ). Carry‐over was tested by injecting a blank sample after a sample spiked at ×10 the concentration of the ULOQ.

As MNE was not commercially available, MNEA was used in its place as a surrogate in calibrators and quality controls. The yield of MNEA was investigated using the analogues pair etiocholanolone and epiandrosterone.

### 2.6 Analysis of Formulation Impurity

One tablet of NorLevo was crushed and extracted by sonicating it in 2 mL methanol for 30 min. The concentration estimation was performed using a single reference point, also in methanol, in triplicate. The same instrument method as above was applied.

### 2.7 Data Analysis

All concentrations were normalized using specific gravity according to the formula: normalized concentration (ng/mL) = concentration (ng/mL) × 0.02 × (SG—1)^−1^.

Plots were produced with R Statistical Software using the ggplot2 package [17, 18].

## 3 Results

### 3.1 Assay Performance

Precision and accuracy were within 15% (20% LLOQ) of the nominal value. Linearity was determined and the quantification ranges were set according to Table 1. Selectivity, specificity, matrix effect and carry‐over were acceptable, with one exception where inaccuracy was observed in one of the matrix effect samples.

The ionisation efficacy was measured to be 30.1% higher for epiandrosterone compared to etiocholanolone. As MNE concentrations were calculated from MNEA calibrators, they were by analogy multiplied by a factor of 1.3.

### 3.2 Analysis of Formulation

Impurities in the NorLevo formulation consist of approximately 12 ng 18MENT.

### 3.3 Study Samples

All participants collected samples with 100% compliance, which resulted in a total of 35 samples.

**FIGURE 3:**
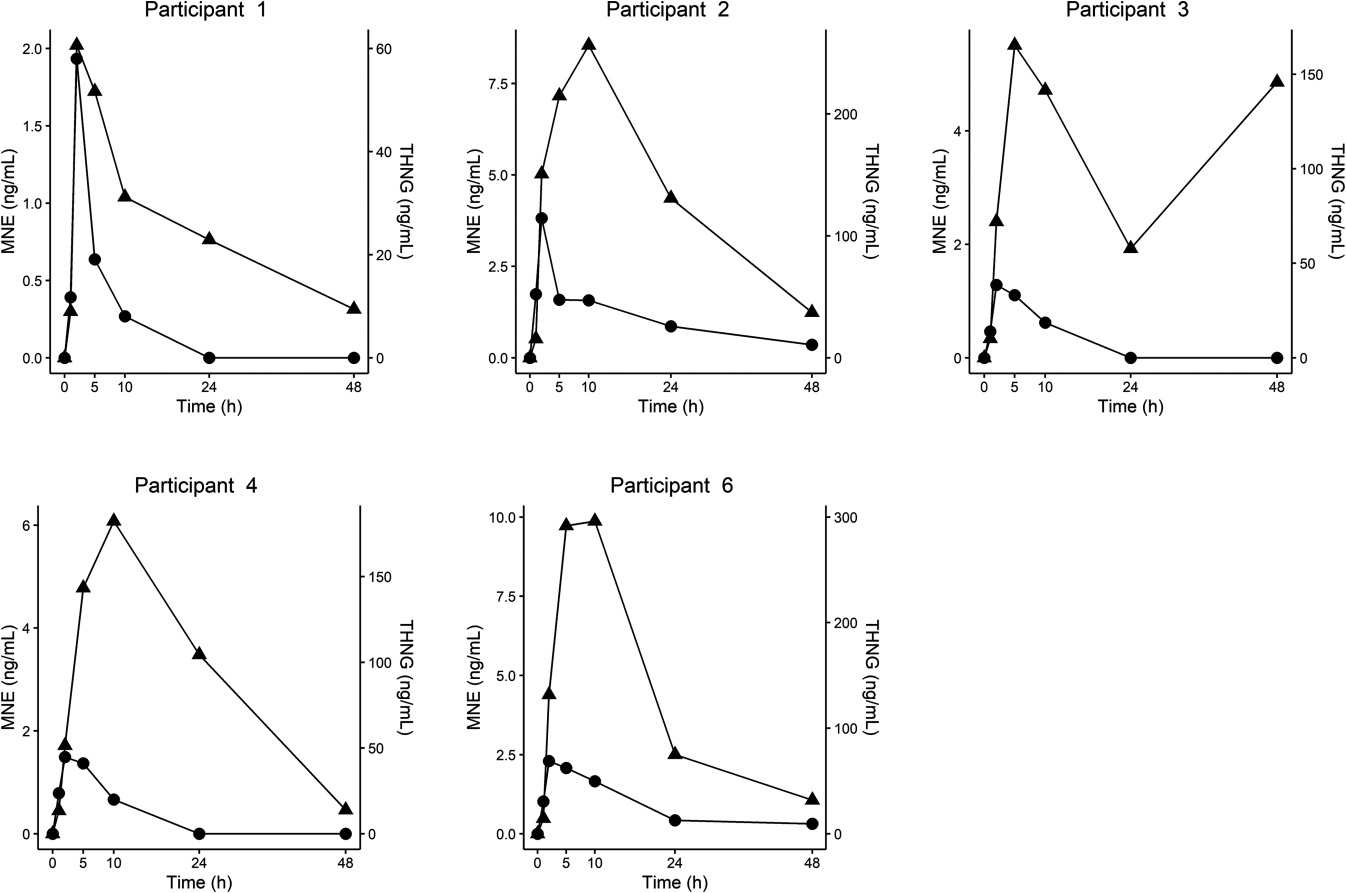
The excretion profile of the prohibited compound 18‐methyl‐19‐noretiocholanolone, MNE (circle, left axis), and emergency contraceptive marker tetrahydronorgestrel, THNG (triangle, right axis), over the course of the study. Concentrations are corrected by specific gravity.

With the exception of 18MENT, all targets in Table 1 could be detected in every participant after administration. These results confirm the crossing metabolic pathways of LNG and 18MENT. The excretion profiles of MNE and THNG can be seen in Figure 3. Inter‐individual variation was observed in the excretion of LNG, MNE and THNG. The maximum concentration (t_max_) for the prohibited metabolite, MNE, was always reached after 2 h, while THNG t_max_ ranged between 2 and 10 h. The highest concentration (C_max_) of the prohibited metabolite was narrow (1.5–2.5 ng/mL) whilst THNG had a much larger span (60–300 ng/mL). LNG had similar characteristics to MNE but could be detected at low levels even in the final sample (48 h) of every participant.

## 4 Discussion

Our results suggest that LNG is the source of the prohibited metabolite MNE detected in urine. MNE is present in all participants urine for at least 1 day after administration. This may have negative consequences for female athletes that throughout their careers could need emergency contraception. In the last year alone, three samples at the Karolinska doping control laboratory have contained the prohibited metabolite (MNE) but could be dismissed by monitoring THNG. Levonorgestrel was also present in all three samples (data not published).

18‐methyl‐19‐nortestosterone was detected at low concentrations in the preparation. This is most likely an impurity from synthesis, similar to what Walker et al. reported for norethisterone, which contained 17–1015 ng of 19‐norandrostenedione [19]. No 18MENT could, however, be detected in any of the participant samples after administration. This was expected and has been observed for similar compounds such as methyltestosterone [20]. The amount of 18MENT in the formulation was not enough to be the single source of MNE.

We have investigated the use of LNG and THNG as potential markers for emergency contraceptive use. Even though the study period was short, participants last sample (48 h) contained substantial amounts of THNG and negligible amounts of MNE. These findings are supported by a study by Littleton where THNG was determined to be the main metabolite. Littleton also reports that the glucuronic conjugates aren't the only metabolites, LNG is excreted as sulphates [11], and further, 50% of LNG is excreted in feces [21]. LNG t_max_ was 2 h (C_max_: 1.5–2.5 ng/mL) in each participant, in line with previous observation [22] which unfortunately decreases its usefulness as a marker for LNG administration. THNG, on the other hand, had a higher t_max_ and C_max_, which greatly extends the detection window.

In the case of norethisterone, health‐care professionals advise athletes against its use. Our results indicate that female athletes could be falsely accused if they use emergency contraceptives containing LNG. With that in mind, emergency contraceptives are normally used within 72 h after sexual intercourse, and LNG is available over the counter in many countries. This limits the freedom of choice, especially if alternatives are limited.

No MNE could be detected when continuous use at lower doses of levonorgestrel (150 μg) was investigated in 55 females [13]. Nonetheless, since levonorgestrel in the form of emergency HC exposes athletes to the risk of an anti‐doping rule violation, it is strongly recommended that anti‐doping laboratories also monitor THNG when confirming 18‐methyl‐19‐nor steroids.

## 5 Conclusion

Monitoring tetrahydronorgestrel adds complexity to results interpretation; however, this is warranted as a marker of levonorgestrel. Laboratories shall consider this to avoid falsely accusing female athletes of doping.

## 6 Limitations

For the intents of this paper, the relative concentrations of the analytes are of interest and therefore measurements below LOQ were included.

## Conflicts of Interest

The authors declare no conflicts of interest.

## Data Availability

The data that support the findings of this study are available on request from the corresponding author. The data are not publicly available due to privacy or ethical restrictions.
